# Complement modulation reverses pathology in Y402H‐retinal pigment epithelium cell model of age‐related macular degeneration by restoring lysosomal function

**DOI:** 10.1002/sctm.20-0211

**Published:** 2020-08-20

**Authors:** Edvinas Cerniauskas, Marzena Kurzawa‐Akanbi, Long Xie, Dean Hallam, Marina Moya‐Molina, Kathryn White, David Steel, Mary Doherty, Phil Whitfield, Jumana Al‐Aama, Lyle Armstrong, David Kavanagh, John D. Lambris, Viktor I. Korolchuk, Claire Harris, Majlinda Lako

**Affiliations:** ^1^ Biosciences Institute, Faculty of Medical Sciences Newcastle University Newcastle upon Tyne UK; ^2^ Clinical & Translational Research Institute, Faculty of Medical Sciences Newcastle University Newcastle upon Tyne UK; ^3^ University of the Highlands and Islands Inverness UK; ^4^ Department of Genetic Medicine and Princess Al‐Jawhara Center of Excellence in Research of Hereditary Disorders, Faculty of Medicine King Abdulaziz University Jeddah Saudi Arabia; ^5^ National Renal Complement Therapeutics Centre, Royal Victoria Infirmary Newcastle upon Tyne UK; ^6^ Department of Pathology and Laboratory Medicine University of Pennsylvania School of Medicine Philadelphia Pennsylvania USA

**Keywords:** autophagy, C3, C3b, C5b‐9, complement activation, complement factor H, human induced pluripotent stem cells, lysosome, retinal pigment epithelium, Y402H polymorphism

## Abstract

Age‐related macular degeneration (AMD) is a multifactorial disease, which is characterized by loss of central vision, affecting one in three people by the age of 75. The Y402H polymorphism in the complement factor H (*CFH*) gene significantly increases the risk of AMD. We show that Y402H‐AMD‐patient‐specific retinal pigment epithelium (RPE) cells are characterized by a significant reduction in the number of melanosomes, an increased number of swollen lysosome‐like‐vesicles with fragile membranes, Cathepsin D leakage into drusen‐like deposits and reduced lysosomal function. The turnover of C3 is increased significantly in high‐risk RPE cells, resulting in higher internalization and deposition of the terminal complement complex *C5b*‐*9* at the lysosomes. Inhibition of C3 processing via the compstatin analogue Cp40 reverses the disease phenotypes by relieving the lysosomes of their overburden and restoring their function. These findings suggest that modulation of the complement system represents a useful therapeutic approach for AMD patients associated with complement dysregulation.

## Graphical abstract

Schematic presentation of autophagy‐lysosomal pathway dysfunction in Y402H‐AMD RPE cells and the impact of Cp40 in restoring the lysosomal function.

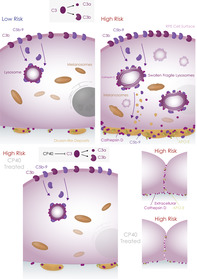

**Significance statement**

Currently, there is no treatment for dry age‐related macular degeneration (AMD), which comprises the majority of AMD pathology. In a collaborative effort, this study describes a novel link between uncontrolled complement activation and autophagy‐lysosome axis, which is caused by increased deposition of the terminal attack complex C5b‐9 at the lysosomes, leading to their overburdening and malfunction. Using an inhibitor of C3 processing, Cp40, this study shows that all the disease phenotypes are reversed, relieving the lysosomes of their overburden and restoring their function. These findings suggest that modulation of the complement system represents a useful therapeutic approach for AMD patients associated with complement dysregulation.

## 1 INTRODUCTION

Maintenance of cellular homeostasis is critical for cellular survival and is achieved through degradation of damaged cellular proteins/organelles and continuous recycling of metabolites. The autophagy‐lysosome pathway plays an important role in maintaining this cellular homeostasis by sequestering unnecessary and damaged cellular components into autophagosomes, which ultimately fuse with lysosomes where their content is degraded.^1^ A block in this system leads to the build‐up of waste material, which has the potential to cause damage and even cell death. The oxygen‐rich environment of the outer retina results in high levels of oxidized waste that needs to be removed to preserve vision. Retinal photoreceptors are highly metabolic cells subjected to light‐induced oxidative stress, which increases the burden of damaged organelles and macromolecules.^2^, ^3^ The homeostasis of photoreceptors is critically dependent on an active autophagy response, that follows the circadian cycle and ensures that damaged macromolecules and metabolites are removed efficiently and cellular organelles (mitochondria, ribosomes, Golgi complex, and outer segments) are repaired.^4^ Retinal pigment epithelial (RPE) cells are exposed to oxidative stress due to their constant exposure to light, high levels of lipid peroxidation products from photoreceptors, and high oxygen utilization. RPE cells phagocytose photoreceptor outer segments (POS) daily and both phagocytosis and autophagy converge into the lysosomes for degradation of their substrates. As such, RPE cells are highly susceptible to lysosomal dysfunction, which in turn has been shown to result in accumulation of large degradation resistant macromolecules, such as lipofuscin, and release of partially digested or undigested material.^2^ Lipofuscin accumulation can increase lysosomal pH and reduce lysosomal degradation, which in turn impairs the functions of RPE leading to RPE and/or photoreceptor loss.^3^, ^5^ For these reasons, any impairment in autophagy could be detrimental to cellular functions of RPE and retinal cells.

Dysregulation of the autophagy‐lysosome pathway has been implicated in a number of inflammatory, neurodegenerative, and age‐related diseases,^6^ including age‐related macular degeneration (AMD). AMD is one of the most common causes of blindness, affecting one in three people by the age of 75. It accounts for 50% of blind and partially sighted registration with an estimated prevalence of ~600 000 significantly visually impaired people in the United Kingdom and over 8 million worldwide.^7^, ^8^, ^9^, ^10^ AMD is a multifactorial progressive disease with a complex interaction between environmental, metabolic, and hereditary factors as well as chronic innate immune activation.^11^ Vision loss in AMD is caused either by apoptosis of the RPE or overlying photoreceptors or both. AMD occurs in two advanced forms: “dry” AMD where cellular debris called drusen accumulates between the choroid and the retina with RPE and photoreceptor cell atrophy, and the “wet” form where neovascularization from the choroid occurs underneath the retina. Treatments are in widespread use for the choroidal neovascularization associated with wet AMD including anti‐VEGF agents^12^, ^13^, ^14^; however, no treatment exists for dry AMD and there is a huge unmet need for investigations into therapies for this disease. The James Lind Alliance priority setting exercise in AMD research identified the creation of treatments to stop the progress of dry AMD as the number one priority in AMD research.^15^

Several lines of evidence suggest an association between dysfunctional autophagy‐lysosome pathway and AMD. For example, increased autophagic flux and higher expression of autophagic key proteins have been observed in retinae of early AMD patients, which may suggest an increased demand for clearance of accumulated damaged organelles and macromolecules at the early stages of the disease.^16^, ^17^ However, the opposite was observed in late stages of AMD, which may reflect an overload or dysfunction of the autophagic system that is unable to cope with the increased demand for clearance of damaged organelles. This is corroborated by findings that key pathogenic features of AMD, namely increased mitochondrial damage, lipid peroxidation and accumulation of N‐retinylidene‐N‐retinyl‐ethanolamine (A2E, a key component of lipofuscin) can result in decreased lysosomal activity in RPE cells and impairment of autophagosome‐lysosome fusion.^17^, ^18^ Autophagic and exosomal proteins are found in drusen, leading to the hypothesis that increased autophagic activity together with lysosomal dysfunction may result in the release of intracellular proteins via exosomes.^17^ Mice deficient in key components of autophagy (*Beclin 1* and *Atg7*) develop severe retinal degeneration upon light exposure^19^ and bi‐allelic mutations in the autophagy regulator *DRAM2* result in development of retinal degeneration with early macular cone photoreceptor involvement, suggesting an important role for autophagy in retinal homeostasis and function.^20^

Despite these associations, it remains unclear whether changes in autophagic flux and function are a cause or a consequence of disease. The paucity of information on the role of autophagy in the pathophysiology of AMD in previous studies was due to the lack of an adequate human in vitro AMD disease model that recapitulates many aspects of this multifactorial disease and can be used as a reliable source to study the role of autophagy in AMD. We have been able to overcome this limitation by developing a physiologically relevant RPE human disease model of AMD caused by the most common risk factor (complement factor H [*CFH*] polymorphism Y402H) that recapitulates key features of AMD and displays an impaired autophagic response.^21^ Recent data suggest that complement activation can contribute to regulation of autophagy‐lysosome pathway in infective and inflammatory diseases.^22^ For example, sublytic C5b‐9 membrane attack triggers lysosomal membrane permeabilization and cathepsin leakage from lysosomes resulting in podocyte injury in idiopathic membranous nephropathy.^23^ C3 protein has also been shown to be expressed intracellularly in pancreatic β cells, where it binds autophagy‐related protein ATG16L1, regulating autophagy and protecting β cells from dying, highlighting a novel intracellular protective role for this important complement regulator.^24^ Interestingly, complement activation of C3 in other cell types (eg, human CD4+ T cells) has been shown to occur via cathepsin L‐mediated cleavage, resulting in C3a mediated stimulation of intracellular C3aR signaling in lysosomes, which sustains homeostatic T‐cell survival.^25^ Collectively this evidence suggests complex links between intracellular complement‐mediated signaling, autophagy, and fluid‐phase complement activation, which is cell type‐dependent.

In this article, we have used the induced pluripotent stem cell derived RPE (iPSC‐RPE) disease model to investigate which steps of autophagy‐lysosome pathway are affected in AMD patients with the Y402H‐*CFH* polymorphism and the interplay between complement activation and the autophagy‐lysosome pathway. Our data suggest that Y402H patient‐specific RPE cells are characterized by increased C3 turnover, which results in increased C5b‐9 deposition within lysosomes, resulting in their swelling and reduced membrane integrity. Inhibition of C3 turnover with the compstatin analogue Cp40 results in the reversal of the RPE cellular phenotypes, the restoration of the lysosomal number, size and function, and a significant reduction in deposition of drusen‐like deposits.

## 2 RESULTS

### 2.1 High‐risk RPE cells are characterized by an expanded and less functional lysosomal compartment and disrupted melanogenesis

In our previous study, we reported a significant increase in LC3 puncta and p62/SQSTM1 aggregates in RPE cells derived from high risk (homozygous for the Y402H‐*CFH* polymorphism) and affected AMD patients compared to those derived from low‐risk (homozygous for the wild type *CFH*) nonaffected subjects, which suggests autophagosome accumulation.^21^ To investigate in detail which step of autophagy is affected in high‐risk RPE cells, we analyzed markers of autophagosome initiation (ATG12‐ATG5) and formation (LC3‐I/II) as well as lysosomal (CTSD, LAMP1, and p62) and late endosomal/exosomal (CD63) markers. In accordance with our previous study, we observed a significant increase in the expression of LC3‐II, p62, and ATG12‐ATG5 (Figure S1A) and LC3 and p62 puncta in RPE cells derived from high‐risk AMD patients (Figure S1B). We also performed autophagy flux experiments, by combining application of a known allosteric mammalian Target of Rapamycin Complex I (mTORC1) inhibitor and autophagy inducer, Rapamycin for 24 hours with Bafilomycin A1 (late stage autophagy inhibitor) treatment for the last 4 hours of treatment. These flux experiments showed a decrease in pS6 expression in both low‐ and high‐risk RPE cells upon application of Rapamycin (Figure S1C). As expected, low‐risk RPE cells showed an increase in LC3‐II expression upon application of Rapamycin, which was further augmented when late stages of autophagy were blocked by Bafilomycin A1 expression; however, this was not the case for high‐risk RPE cells, where combined application of Rapamycin and Bafilomycin did not augment LC3‐II expression compared to Rapamycin treatment alone (Figure S1C). Together, these data suggest reduced autophagic flux in the high‐risk RPE cells.

**FIGURE 1:**
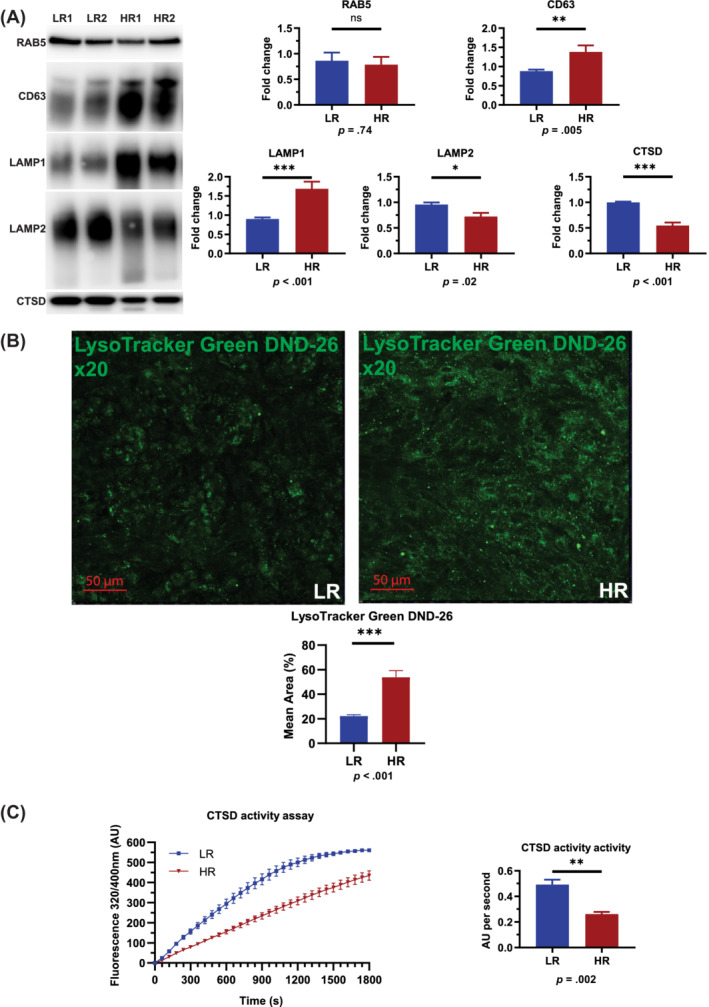
Assessment of lysosomal marker expression in low‐ and high‐risk RPE cells. A, Western blotting quantification shows no changes in RAB5 expression, a significant increase of LAMP1 and CD63 and a significant decrease of CTSD, pro‐CTSD, and LAMP2 expression. All quantification data were normalized to the total protein blots and shown as fold change against low‐risk RPE cells. Data shown as mean ± SEM (data from at least six replicates); B, Fluorescence microscopy analysis shows a significant increase of acidic vesicles detected through LysoTracker Green DND‐26 in high‐risk RPE cells. Data shown as mean ± SEM (data from at least six replicates); C, Cathepsin D activity assay shows a significant decrease of activity in high‐risk compared to low‐risk RPE cells. The activity assays data were normalized against low‐risk RPE cells and shown as fold change. Data shown as mean ± SEM (data from three replicates). Statistical significance was assessed using an unpaired *t* test. HR, high‐risk retinal pigment epithelium cells; LR, low‐risk retinal pigment epithelium cells; RPE, retinal pigment epithelium

LC3 is a constitutive component of autophagosomal membranes, while p62 is a target of autophagy degradation. Increased expression of both markers in high‐risk RPE cells suggests a block in the late stages of the autophagy‐lysosome pathway; hence, we proceeded with analysis of markers involved in the degradation process. We did not see a difference in expression of early endosome marker RAB5 between low‐ and high‐risk RPE cells (Figure 1A), however, LAMP1 and CD63 expression was significantly increased in high‐risk RPE cells (Figure 1A), suggesting an expansion of the late endosomal/lysosomal compartment and/or increased exocytosis. These findings were further corroborated by increased Lysotracker staining in high‐risk RPE cells (Figure 1B). Interestingly, LAMP2 expression was significantly reduced in high‐risk RPE cells, suggesting an impaired lysosomal maturation in high‐risk RPE cells (Figure 1A). The expression of the key lysosomal enzyme, Cathepsin D, and its activity were significantly reduced (Figure 1A,C) in high‐risk RPE cells. Together these data suggest that high‐risk RPE cells are characterized by an expanded, albeit less functional lysosomal compartment.

**FIGURE 2:**
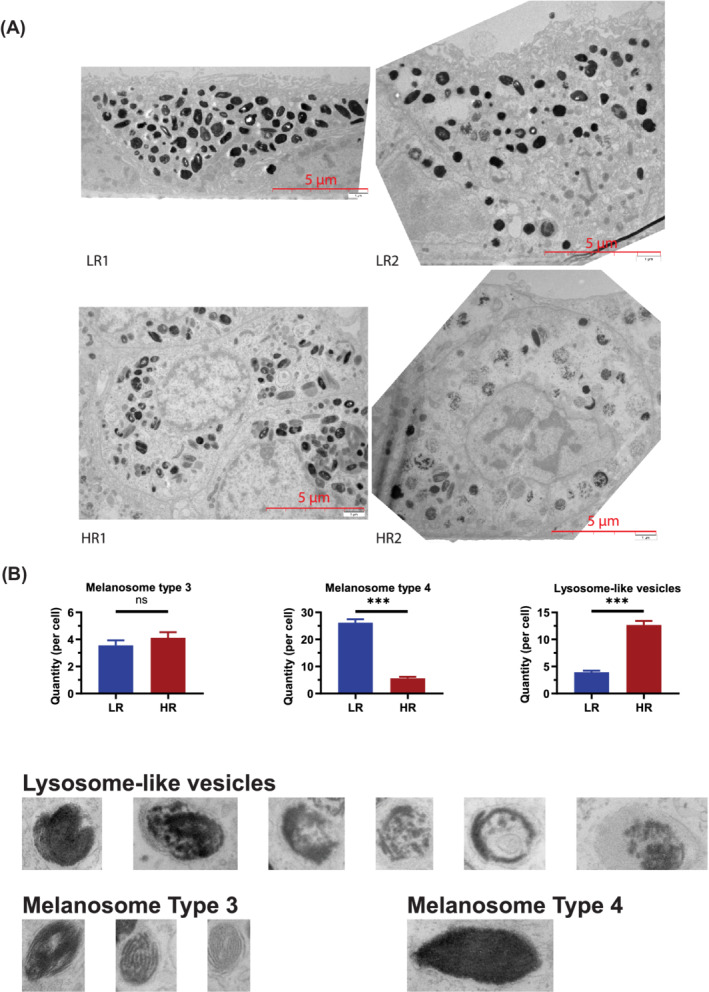
TEM analysis shows reduced number of melanosomes and increased number of lysosome‐like vesicles in high‐risk RPE cells. A, Representative TEM images of low‐ and high‐risk RPE cells and examples of melanosomes types 3, 4 and lysosome‐like‐vesicles, which were counted from at least 10 cells from each high and low‐risk RPE; B, Graphical representation of quantification analysis showing reduced number of melanosomes and increased number of lysosome‐like vesicles in high‐risk RPE cells. Data shown as mean ± SEM, n = 10 to 15. Statistical significance was assessed using an unpaired *t* test, ***P < .001. HR, high‐risk retinal pigment epithelium cells; LR, low‐risk retinal pigment epithelium cells; RPE, retinal pigment epithelium; TEM, transmission electron microscopy

We performed transmission electron microscopy (TEM) analysis to quantify the number of melanosomes and lysosomes. We observed the presence of melanosomes type 3 (where the lamellae are not tightly compacted) and mature melanosomes type 4 (where the melanin deposits are thick preventing visibility of fibrils in the internal structure^26^) in both low‐ and high‐risk RPE cells (Figure 2A). Low‐risk RPE cells contained a large number of type 4 melanosomes and a small number of lysosome‐like‐vesicles. We did not observe significant changes in the number of type 3 melanosomes between low‐ and high‐ risk RPE cells; however, the number of type 4 melanosomes was significantly reduced and lysosome‐like‐vesicles was significantly increased in high‐risk RPE cells (Figure 2A,B). Unlike melanosome synthesis in the skin, where melanosome biogenesis occurs continuously, in RPE cells this process is completed before birth and pigment granules are retained throughout life^27^; hence, a reduction in mature melanosome numbers in high‐risk RPE cells is likely to compromise their ability to absorb stray light. Defects in melanosome biogenesis and movement are associated with human retinal disease, and a deficit of melanin pigment in the RPE is associated with aging and AMD. Our data corroborate these findings and demonstrate disrupted melanogenesis in high‐risk RPE cells.

Collectively these data suggest the most affected step of autophagy‐lysosome pathway in high‐risk RPE cells is at the lysosomes, which despite an increase in number are unable to mature and process the last stage of autophagy due to a reduction in Cathepsin D expression and activity, preventing autophagosome maturation and acidification.

### 2.2 Inhibition of mTORC1 by Rapamycin application has no beneficial impact on high‐risk RPE cells

Published studies have shown that activation of lysosomal function can be achieved by suppressing mTORC1 activity.^28^ Seven day Rapamycin treatments were carried out in high‐risk RPE cells to assess the impact on lysosomal number and ultrastructure. There were no significant changes in Cathepsin D activity assays in high‐risk RPE cells in response to Rapamycin treatment (Figure S2A). The number of lysosome‐like vesicles was unchanged in Rapamycin treated high‐risk RPE cells (Figure S2B,C); however, a significant increase in the number of stress vacuoles was observed (Figure S2D). Together these data suggest that application of Rapamycin has no beneficial impact on the high‐risk RPE cells, thus corroborating the clinical trials of mTORC1 inhibitor^29^ (sirolimus), which have shown no evidence of anatomical or functional benefit in treated eyes of patients with geographic atrophy. Furthermore, one of the trials suggested that application of sirolimus might potentially be associated with effects detrimental to visual acuity, which could be due to increased cell stress observed in this study in high‐risk RPE cells in response to Rapamycin treatment.

### 2.3 High‐risk RPE cells are characterized by Cathepsin D deposition in drusen‐like deposits and fragility of lysosomal membranes

**FIGURE 3:**
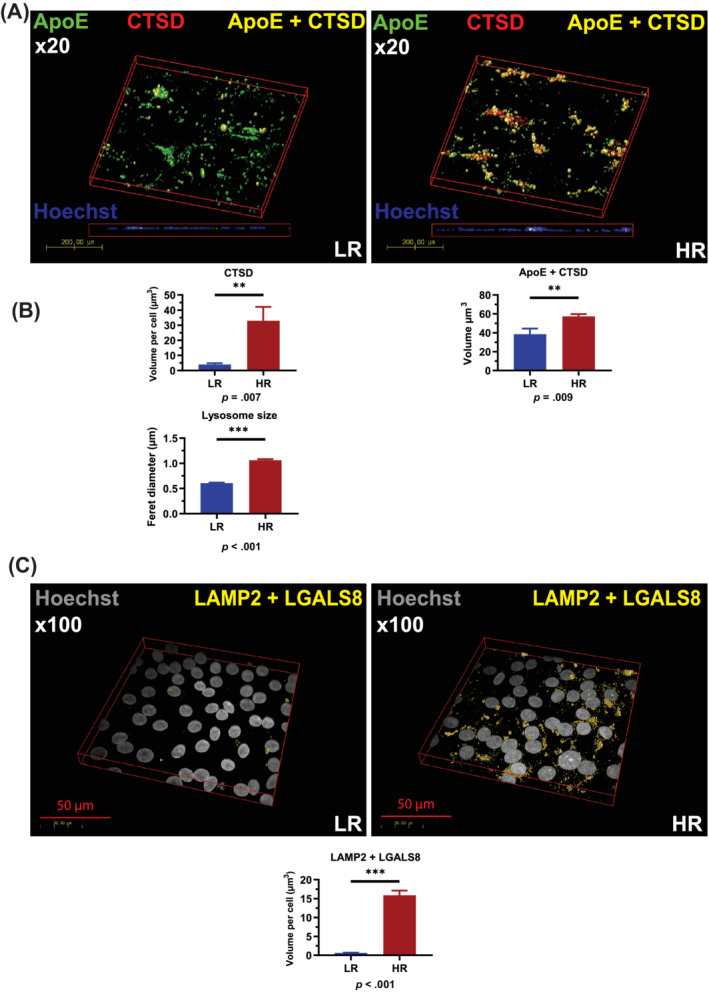
High‐risk RPE cells are characterized by fragile lysosomal membranes and deposition of Cathepsin D into drusen‐like deposits. A, Immunocytochemistry data show a significant increase of extracellular CTSD per cell in high‐risk RPE cells and a significant increase of ApoE/CTSD colocalized volume, suggesting deposition of Cathepsin D into drusen‐like deposits; B, TEM images were used to measure lysosome‐like vesicle size, which is clearly increased in high‐risk RPE cells, data shown as mean ± SEM (measurements performed in at least 10 cells from each high and low‐risk RPE); C, Increased lysosomal membrane fragility shown by increased colocalization of LGASL8 with LAMP2 expression. A,C, Data were calculated as volume/cell. Data shown as mean ± SEM (data from at least six different images per cell line, all experiments performed in biological triplicates). Statistical significance was assessed using an unpaired *t* test. HR, high‐risk RPE; LR, low‐risk RPE; RPE, retinal pigment epithelium; TEM, transmission electron microscopy

RPE cells express high levels of Cathepsin D, which has been shown to play an important role in the breakdown and clearance of POS; hence, we investigated further the impact of reduced Cathepsin D expression and activity in high‐risk RPE cells. Mice that express an enzymatically inactive form of Cathepsin D develop RPE hypopigmentation, shortening of POS, photoreceptor death, and accumulation of basal and laminar deposits, which are considered to be a key feature of AMD.^30^ Studies performed in human AMD eyes have also shown a small increase in Cathepsin D immunoreactivity around hyalinized drusen.^31^ We therefore investigated the presence of Cathepsin D immunoreactivity in drusen‐like deposits using a previously described porous membrane transwell RPE culture, which generates an apical chamber corresponding to the retinal facing side and the basal chamber corresponding to the choroidal facing side of the RPE, where drusen deposits exist.^32^ Immunofluorescence analysis of Cathepsin D with Apolipoprotein E (ApoE), a known marker of drusen deposits, indicated a significantly higher accumulation of extracellular Cathepsin D in deposits localized between and under the RPE cells and in particular those marked by ApoE expression (Figure 3A), suggesting a higher Cathepsin D accumulation in drusen‐like deposits in high‐risk RPE cells.

A previous report has indicated that an enlargement of lysosomal vesicles and/or an increase in lysosome numbers precedes lysosomal membrane permeabilization.^33^ In accordance with this, TEM based measurements indicated a significant increase in lysosomal vesicle size in high‐risk RPE cells (Figure 3B). In the Royal College of Surgeons rat model of retinal degeneration, the release of Cathepsin D from the RPE cells is associated with the fragility of lysosomal membranes.^34^ In view of these findings, we performed staining with Galectin 8 (LGALS8), which recognizes lysosomal membrane damage in combination with LAMP2 lysosomal marker. Our data show that Galectin 8 staining in lysosomes is significantly higher in high‐risk RPE cells when compared to low‐risk unaffected controls^35^ (Figure 3C), indicating the fragility of lysosomal membranes, which is the most likely reason for Cathepsin D deposition in sub RPE drusen‐like deposits.

### 2.4 High‐risk RPE cells are characterized by higher C3 turnover, C5b‐9 internalization, and deposition in the lysosomes

In aged mice, depletion of C3 has been shown to ameliorate autophagic activity, the age‐dependent decrease of retinal thickness and function as well as increase the expression of antioxidant enzymes and several key genes that are important for retinal electrophysiological functions, while lowering the expression of cellular oxidative stress markers.^36^ In view of this evidence and our data showing lysosomal swelling and membrane fragility and Cathepsin D leakage from lysosomes, we assessed expression of secreted and intracellularly located key components of C3 signaling pathway. C3 plays a key role in the complement activation as its cleavage results in generation of C3a, which possesses anaphylatoxic as well as immunoregulatory properties, and C3b that promotes complement activation and the subsequent formation of the membrane attack complex. Quantitative reverse transcription polymerase chain reaction (RT‐PCR) analysis indicated a significantly lower expression of *C3*, *C7*, and *C9* and higher expression of *C6* in high‐risk RPE cells (Figure S3A). Native (comprising both C3a and C3b domains) and activated C3 (C3b, iC3b, C3c) were measured in cell supernatants (Figure S3B,C); there was more C3 in the supernatant of low‐risk RPE cells, although proportionally more of the C3 was converted to fragments as indicated by the ratio of fragments:C3 in high‐risk RPE cells (Figure S3D).

**FIGURE 4:**
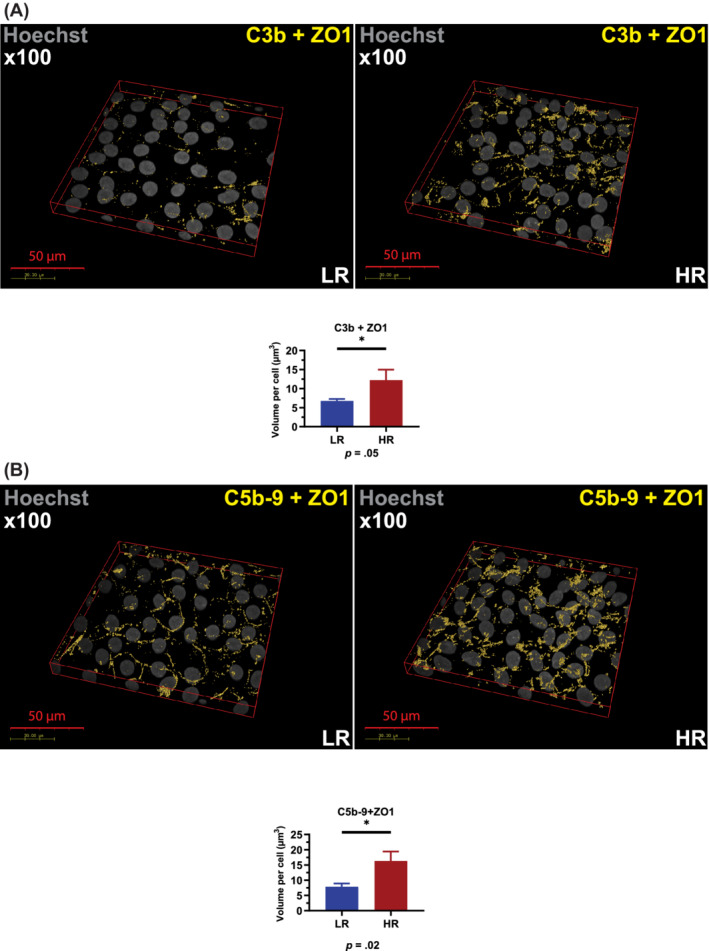
High‐risk RPE cells are characterized by increased C3b and C5b‐9. A, Immunocytochemistry data show a significant increase of C3b associated with high‐risk RPE cells; B, Immunocytochemistry data show a significant increase of C5b‐9 associated with RPE cells. A,B, Data were calculated as volume/cell. Data shown as mean ± SEM (data from at least six different images per cell line, all experiments performed in biological triplicates). Statistical significance was assessed using an unpaired *t* test. HR, RPE; LR, low‐risk RPE; RPE, retinal pigment epithelium

Next, we went on to investigate the C3b on the cell membrane by confocal microscopy using an antibody that recognizes a neo‐epitope expressed on the cleavage fragments of C3b, iC3b, and C3c (Figure 4A). We used colocalization with ZO‐1, a tight junction protein located on the cell membrane of RPE cells^37^ to assess complement component association with RPE cells. This analysis indicated a significant increase of C3b associated with high‐risk RPE cells when compared to low‐risk control (Figure 4A), despite the lower level of C3 secretion. It is possible that lower levels of soluble, native C3 (Figure S3B) reflect more effective membrane activation and thus cellular location of C3.

**FIGURE 5:**
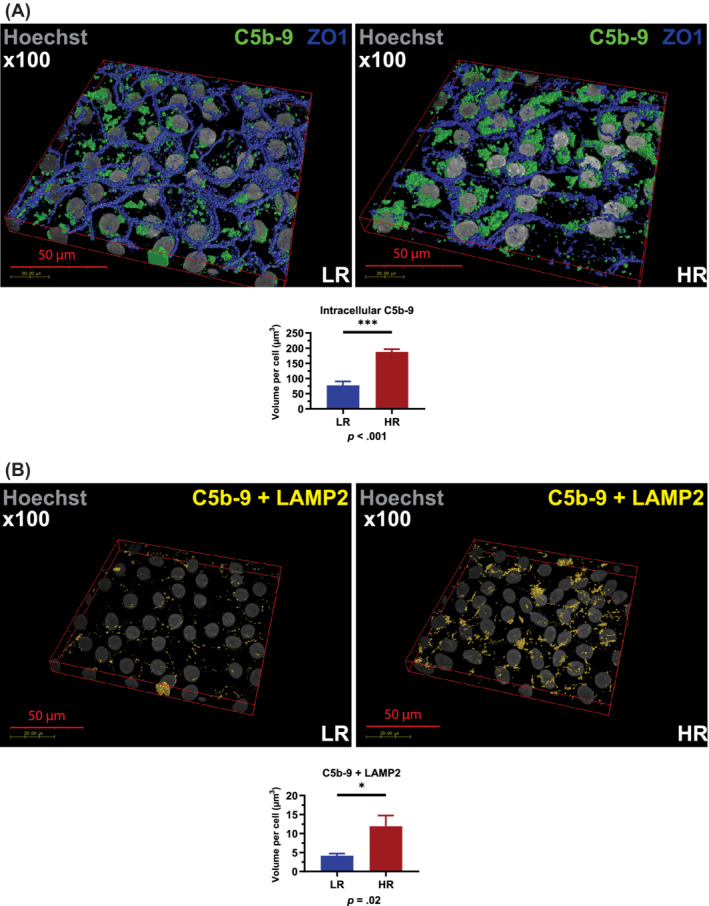
High‐risk RPE cells are characterized by increased levels of intracellular and lysosome‐localized C5b‐9. A, Immunocytochemistry data show a significant increase of C5b‐9 intracellular localization; B, Immunocytochemistry data show a significant increase of C5b‐9 localization at the lysosomes assessed by colocalization with LAMP2. A,B, Data were calculated as volume/cell. Data shown as mean ± SEM (data from at least six different images per cell line, all experiments performed in biological triplicates). Statistical significance was assessed using an unpaired *t* test. HR, high‐risk RPE; LR, low‐risk RPE; RPE, retinal pigment epithelium

Proteolytic cleavage of C5 by C5 convertase generates C5b, which initiates assembly of the C5b‐9 complex. The last step of C5b‐9 complex formation involves polymerization of C9, which accompanies insertion of the complex into the cell membrane. Deposition of C5b‐9 terminal complement complexes in association with the Apo‐E rich sub‐RPE deposits is a key feature of AMD. Recent published work suggests that RPE cells are able to mitigate the effect of complement attack by endocytosis of C5b‐9 and processing through the lysosomes.^38^ Given the higher C3 turnover in high‐risk RPE cells, we hypothesized that more C5b‐9 deposition would occur on the surface of high‐risk RPE cell leading to higher internalization and localization within lysosomes. Immunofluorescence data showed a significantly increased colocalization of C5b‐9 with ZO‐1 (Figure 4B) and a significantly higher intracellular C5b‐9 accumulation in the high‐risk RPE when compared to low‐risk control cells (Figure 5A), suggesting possible endocytosis of excess C5b‐9 from the cell membranes. We used the colocalization of C5b‐9 with LAMP2 as an indicator of internalized complement proteins within the more mature lysosomes. This analysis showed a significantly increased C5b‐9 at the lysosomes in the high‐risk RPE cells (Figure 5B). Together these data suggest increased C3 turnover in high‐risk RPE cells, which leads to increased deposition of C5b‐9 associated with RPE cells, followed by enhanced internalization and overloading of the lysosomes.

### 2.5 Inhibition of C3 with compstatin analogue Cp40 results in reversal of cellular phenotypes, restoration of lysosomal function, and reduction of drusen‐like deposits in high‐risk RPE cells

**FIGURE 6:**
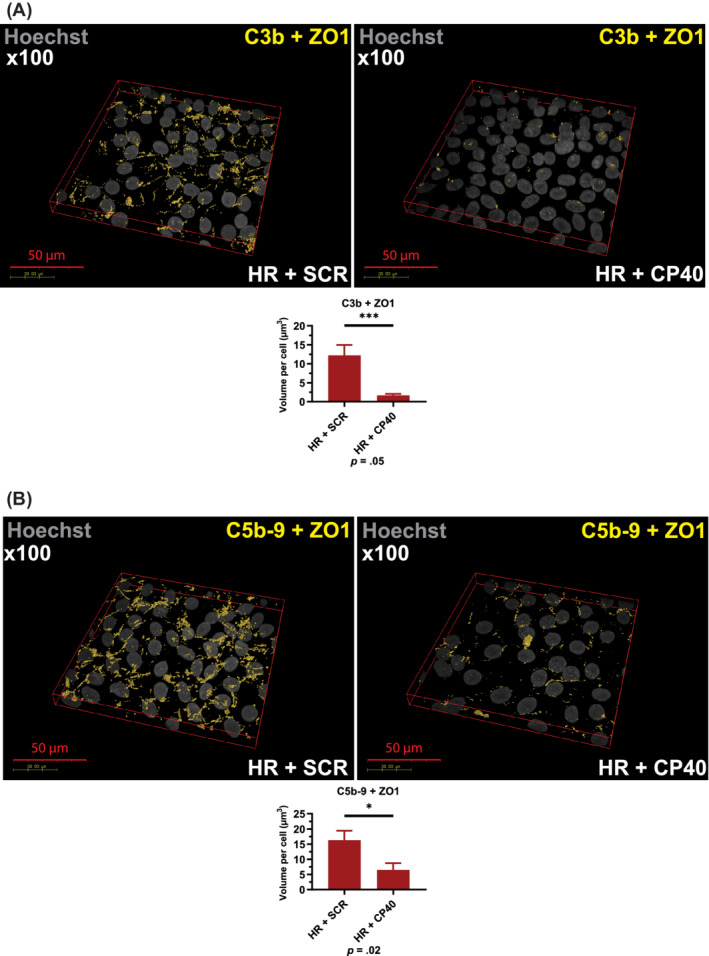
Cp40 treatment reduces C3b and C5b‐9 associated with RPE cells. A, Immunocytochemistry data show a significant decrease of C3b associated with RPE cells upon Cp40 treatment; B, Immunocytochemistry data show a significant decrease of C5b‐9 associated with RPE cells upon Cp40 treatment. A,B, Data were calculated as volume/cell. Data shown as mean ± SEM (data from at least six different images per cell line, all experiments performed in biological triplicates). Statistical significance was assessed using an unpaired *t* test. HR, high‐risk RPE; RPE, retinal pigment epithelium; SCR, scrambled peptide

**FIGURE 7:**
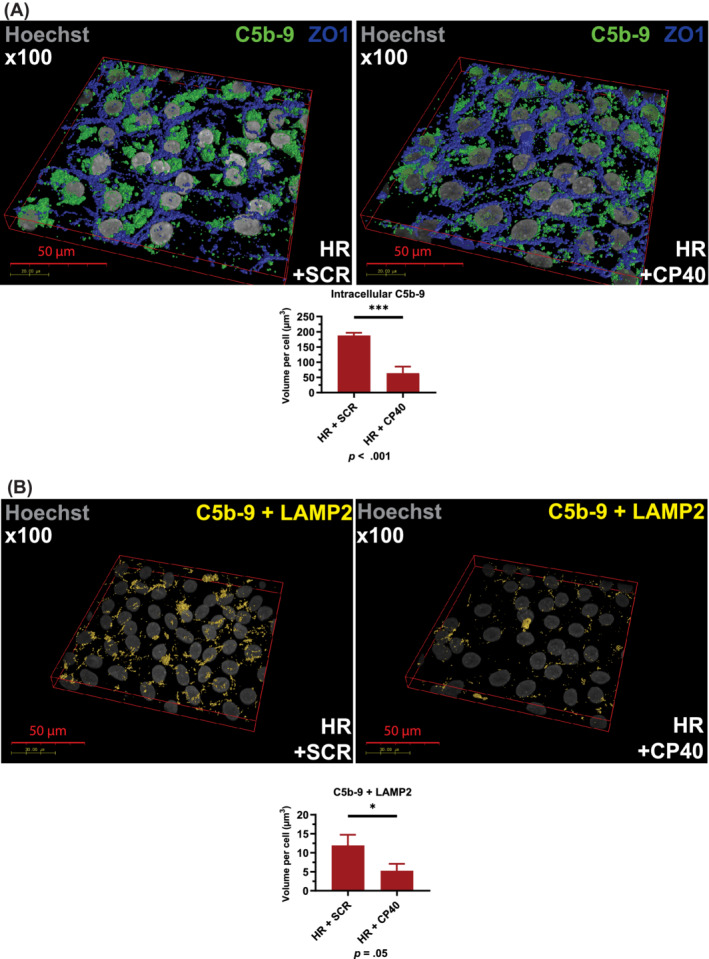
CP40 treatment reduces intracellular and lysosomal C5b‐9 deposition. A, Immunocytochemistry data show a significant decrease of intracellular C5b‐9 upon Cp40 treatment. Data were calculated as volume/cell. Data shown as mean ± SEM (data from at least six different images per cell line, all experiments performed in biological triplicates); B, Immunocytochemistry data show a significant decrease of C5b‐9 localized to lysosomes assessed by colocalization with LAMP2. Data were calculated as volume/cell. Data shown as mean ± SEM (data from at least six different images per cell line, all experiments performed in biological triplicates). Data shown as mean ± SEM (data from at least six different images per cell line). Statistical significance was assessed using an unpaired *t* test. HR, high‐risk retinal pigment epithelium cells; SCR, scrambled peptide

**FIGURE 8:**
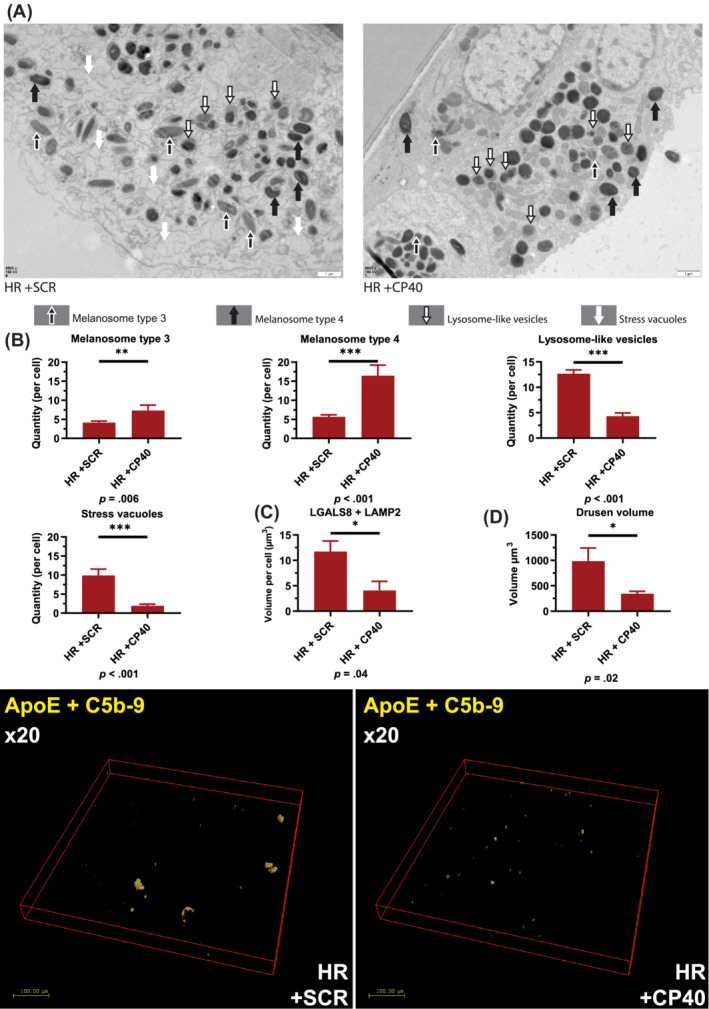
Modulation of complement activity via application of CP40 restores the melanosome and lysosome numbers in high‐risk RPE cells and reduces the deposition of drusen‐like deposits. A, Representative TEM images of high‐risk RPE cells treated with scramble and Cp40 peptides with examples of melanosomes types 3, 4, and lysosome‐like vesicles, which were counted from at least 10 cells from each high‐ and low‐risk RPE cells; B, Graphical representation of quantification analysis showing a significant increase in the number of melanosomes and reduction in the number of melanosomes upon treatment of high‐risk RPE cells with Cp40. Data shown as mean ± SEM, n = 10 to 15; C, Graphical representation showing a significant reduction in membrane fragility assessed by colocalization of LGALS8 with LAMP2. Data were calculated as volume/cell and further normalized to RPE cells treated with scrambled peptide; D, Graphical representation showing a significant reduction in volume of drusen‐like deposits in high‐risk RPE cells following treatment with Cp40. Data were calculated as volume/cell. Data shown as mean ± SEM (data from at least six different images per cell line, all experiments performed in biological triplicates). Statistical significance was assessed using an unpaired *t* test. HR, high‐risk RPE; SCR, scrambled peptide; TEM, transmission electron microscopy

The data presented above suggest increased localization of C5b‐9 in lysosomes of high‐risk RPE cells. This evidence, together with the inability of autophagy inducers to reverse the cellular phenotype in high‐risk RPE cells, led us to hypothesize that uncontrolled C3 turnover is the upstream cause leading to dysregulation of autophagy‐lysosome pathway in high‐risk RPE cells. To this end, we applied the compstatin analogue Cp40,^39^ a cyclic peptide of 14 amino acids with strong and exclusive affinity for human C3 and its fragments C3b/iC3b/C3c. Binding of compstatin to C3 prevents C3 conversion to C3b, which is essential for the functioning of all initiation, amplification, and terminal pathways of complement.^39^, ^40^, ^41^ High‐risk RPE cells were incubated for 7 days with Cp40 or the scrambled control peptide. Quantitative RT‐PCR analysis indicated a significant decrease in the expression of *C3*, *C5*, *C6*, *C7*, and *C8g* in response to Cp40 treatment (Figure S4A). ELISA‐based analysis showed a decreased C3 turnover reflected in the lower ratio of C3 fragments to native C3 (Figure S4B‐D) in the Cp40 treated samples compared to scramble controls. Fluorescence microscopy analysis also indicated reduced C3b (Figure 6A) and C5b‐9 associated with Cp40 treated high‐risk RPE cells (Figure 6B). In addition, the intracellular and lysosomal C5b‐9 expression was significantly reduced (Figure 7A,B) in the high‐risk RPE cells in response to Cp40 treatment. This was associated with increased CTSD expression and Cathepsin D activity (Figure S5A,B) and decreased lysosome size in Cp40 treated high‐risk RPE cells (Figure S5C). TEM analysis showed significant improvements in RPE ultrastructure with Cp40 treatment increasing the number of type 4 melanosomes and reducing the number of lysosome‐like vesicles and stress vacuoles (Figure 8A,B). Most importantly, Cp40 treatment reduced the fragility of lysosomal membranes (Figure 8C) and the volume of drusen‐like deposits in high‐risk RPE cells (Figure 8D).

## 3 DISCUSSION

Our group^21^ and others have successfully applied the iPSC approach to generate disease models for AMD.^42^ These have helped to identify and/or validate the disease risk alleles^43^ generated by genome‐wide association studies^42^ and to highlight key cellular phenotypes in AMD‐RPE related to reduced defense against oxidative damage,^44^ mitochondrial DNA damage and disintegration,^45^ higher expression of complement and inflammatory markers,^46^ dysregulated autophagy,^16^, ^21^, ^47^ and activation of inflammasome signaling.^48^ Focusing on a key high‐risk polymorphism (Y402H) in the complement factor gene, we have used the iPSC‐derived RPE cells to identify which step(s) of autophagy‐lysosome pathway is (are) affected in AMD patients, how it is controlled and test whether it can be reversed. Data obtained during this study indicate that AMD‐RPE cells are characterized by an increased number of dysfunctional and swollen lysosome‐like‐vesicles with fragile membranes, which may lead to deposition of key components of lysosomal content into the drusen‐like deposits and disrupted melanogenesis. Our data show increased C3 turnover in high‐risk RPE cells, which leads to higher C3b and C5b‐9 associated with RPE cells, in turn resulting in an increased internalization of C5b‐9 and overloading of the lysosomal compartment (for a schematic summary, refer to the *graphical abstract*). Inhibition of C3 conversion to C3b via application of compstatin analogue Cp40 reduces C3 turnover and C5b‐9 deposition in the lysosomes as well as lysosome‐like‐vesicle number, size, swelling, and membrane fragility. Cp40 treatment also results in an increased in Cathepsin D expression and activity as well as a significant reduction in the volume of drusen‐like deposits. On the contrary, activation of autophagy by Rapamycin had no beneficial effect in AMD‐RPE cells. Together our findings suggest that lysosomal dysfunction is directly linked to uncontrolled complement activation in AMD, which can be reversed using a small peptide that inhibits the central component of the complement cascade, C3.

Fragility of lysosomal membranes and leakage of their contents into the cytoplasm represents a harmful event for the cells; hence, protective surveillance mechanisms to recognize membrane damage have been developed through binding of the Galectin family of proteins to the cytoplasmic face of the vesicles.^49^ Our data obtained via a combination of methods indicate the presence of an expanded yet dysfunctional lysosomal compartment characterized by lysosome swelling and membrane fragility in high‐risk RPE cells, which can be detected by colocalization of Galectin 8 with lysosomal markers. Galectin 8 has been shown to associate with the mTORC1 apparatus on the lysosome leading to its inhibition and activation of autophagy as shown by the increase in LC3 puncta^50^ and LC3 lipidation. This is very much in accordance with our data showing an increase in LC3 and p62 expression in high‐risk RPE cells, suggesting that the increase in autophagic markers may occur in response to lysosomal membrane damage. This activation of autophagy may also lead to increased “attack” on melanosomes, which in the absence of melanosome biogenesis can result to decreased melanosome numbers as observed in the high‐risk RPE cells. This, however, can be rescued by Cp40 treatment, which alleviates lysosomes of their burden in processing C3b and C5b‐9 fragments, presumably resulting in restoration of autophagic activity and maintenance of melanosome numbers. Melanosome dysfunction/abnormalities are linked to primary lysosome dysfunction in several inherited disease, including Chediak‐Higashi and Hermansky‐Pudlak syndrome.^51^ Hence the disrupted melanogenesis described herein, is very likely to be a consequence of lysosome dysfunction in high‐risk RPE cells.

It is conceivable that induction of autophagy would serve to remove damaged lysosomes in a process named lysophagy.^52^ This process would require ubiquitination of damaged lysosomes, recruitment of autophagic proteins and incorporation into autolysosomes for degradation. This scenario supports the increase in the number of lysosomes and recruitment of autophagic markers to the damaged lysosomal membranes, which is corroborated by the observed colocalization of p62 with LGALS8 (data not shown) in high‐risk RPE cells. This lysosome damage‐driven activation of autophagy is however insufficient to complete the removal and waste degradation due to multiple deficiencies ranging from fragile lysosomal membranes to reduced Cathepsin D expression and activity. In line with this, application of Rapamycin fails to restore the cellular deficiencies in high‐risk RPE cells. We suggest that under these conditions, “burdened” lysosomes may spill their content into the drusen‐like deposits.

What are the possible reasons for damaged lysosomes in RPE cells derived from Y402H‐AMD affected patients? In the last few years, emerging evidence points toward an important role for intracellular C3 in regulation of autophagy, proteasome function and formation of drusen in RPE cells. Several clear examples are provided by recent publications, which show that cholesterol mediated activation of acid sphingomyelinase leads to the increase in cellular ceramide,^53^ which triggers aberrant endosome biogenesis resulting in increased C3a internalization, aberrant mTORC1 activation, and decreased autophagosome biogenesis in RPE cells of an *Abca4* ^−/−^ mouse model of Stargardt early onset with macular degeneration.^54^ Furthermore, the C3a fragment has been shown to induce the formation of sub‐RPE deposits via inhibition of the complement‐driven proteasome inhibition.^55^ In accordance with these studies, we have found an increased number of lysosomes with fragile membranes and enlarged size in RPE cells generated from Y402H‐AMD as well as an increased C3b and C5b‐9 associated with RPE cells, C5b‐9 internalization, and overloading of the lysosomes, which we hypothesize leads to the lysosomal damage and formation of drusen‐like deposits. All these phenotypes are restored upon Cp40 application, indicating that the complement‐associated‐lysosomal damage in high‐risk RPE cells is directly linked to increased C3 turnover in the high‐risk RPE cells.

Both C3 activation and depletion have been associated with retinal degeneration,^56^, ^57^ indicating that careful control of C3 expression and its cleavage fragments needs to be considered for AMD therapies. In view of these studies, we generated iPSC lines with stably integrated shRNAs to *C3*, which resulted in ~85% reduction in *C3* mRNA levels and C3 secretion into the supernatant of high‐risk RPE cells (data now shown). TEM analysis indicated that *C3* knockdown did not restore the number of melanosomes and lysosomes, but most importantly had a detrimental effect in the cells as it increased the number of stress vacuoles, which can lead to cell bursting and death (Figure S6). In contrast to this, inhibition of C3 via the compstatin analogue Cp40 restored the number of lysosomes and melanosomes and reduced lysosomal membrane damage, deposition of drusen‐like deposits and C5b‐9 internalization. Cp40 prevents the conversion of C3 to C3b, which is essential for the initiation, amplification, and formation of the membrane attack complex. CFH and FHL‐1 act as cofactors for CFI and work together to prevent the formation of C3 convertase (C3bBb), which amplifies the generation of C3b. Our data indicate that inhibition of C3 conversion to C3a and C3b restores the cellular phenotypes, lysosomal function and reduces drusen‐like deposits in RPE cells from AMD patients with Y402H polymorphism.

A considerable number of peptides, small molecules, proteins, and antibodies designed to interfere with the complement cascade at various stages of activation have been or are currently being tested in clinical trials.^58^, ^59^ Eculizumab and LFG316, which are monoclonal antibodies that target C5 and prevent its activation, have shown no reduction in the progression of geographic atrophy compared to controls.^58^, ^59^ Similarly, targeting of factor D via Lampalizumab has shown no clinical benefits to patients with AMD in two duplicate large‐scale trials.^58^, ^59^ However, trials focusing on C3 interference have shown results that are more promising. For example, intravitreal injection of the C3 inhibitor compstatin (POT‐4) injection, weekly for 6 months, resulted in drusen disappearance in a primate model with early onset macular degeneration,^60^ while a phase II study with the compstatin analogue (APL‐2, which is the PEGylated version of POT‐4; Apellis Pharmaceuticals) has shown a 29% significant reduction in geographic atrophy growth at 12 months in the monthly intravitreal injection group. Our data with Cp40 application and restoration of normal cellular phenotypes and drusen reduction suggest that Cp40 injections in AMD patients could prove beneficial. To date, Cp40 has shown promising therapeutic efficacy in nonhuman primate models of periodontitis, hemodialysis, kidney transplantation, and hemorrhagic shock.^61^ A successful single phase I trial with Cp40‐based drug candidate AMY‐101 (Amyndas Pharmaceuticals) has been recently completed in healthy male volunteers (NCT03316521) showing very good safety profile (https://www.amyndas.com/amyndas-pharmaceuticals-announces-positive-results-from-a-phase-i-trial-of-its-new-complement-c3-inhibitor-amy-101/). Collectively these findings suggest that inhibition of C3 turnover with Cp40‐based drug candidates like AMY‐106 with more than 3‐month eye residence time may provide an effective therapy for reduction of geographic atrophy in AMD patients with Y402H polymorphism. Further studies looking at the route and frequency of delivery, as well as safety and efficacy are needed to implement these research findings into treatments for AMD.

## 4 MATERIALS AND METHODS

### 4.1 Cell culture

#### 4.1.1 *iPSC culture*

iPSCs were cultured in Matrigel Growth Factor Reduced Basement Membrane Matrix (Corning) coated plates and mTeSR1 (STEMCELL Technologies) growth media in humidified, 5% CO_2_, 37°C environment. iPSCs were passaged in ratio 1:6 every 4 to 6 days using Versene (EDTA) 0.02% (Lonza).

### 4.2 iPSC differentiation to RPE cells

iPSCs were grown to confluence and maintained for two additional days before proceeding with spontaneous differentiation using RPE media (Advanced RPMI (ThermoFisher), 10% knockout serum replacement (ThermoFisher), 2% B27 supplement (ThermoFisher), 1% GlutaMAX (ThermoFisher), 1% Penicillin‐Streptomycin (ThermoFisher). The media was then partially replaced every 2 to 3 days until pigmented cell patches appeared. The pigmented patches were surgically excised, disassociated using TrypLE Select Enzyme (10X) (ThermoFisher) and seeded on Matrigel‐coated plates in RPE media.

### 4.3 iPSC‐RPE culture

iPSC‐RPE were maintained in humidified, 5% CO_2_, 37°C heated incubators. Routine cell maintenance was performed by partially replacing media every 2 to 4 days with RPE media as described by Hallam et al.^21^ No human serum/plasma or indeed calf serum was used in the RPE culture. Cells were passaged in a ratio 1:3 using TrypLE Select Enzyme (10X) and plated on Matrigel Growth Factor Reduced (GFR) Basement Membrane Matrix (Corning) in RPE media. RPE cells were used for experiments up to passage 5 to 6. For final experiments, RPE cells were plated on Matrigel coated 12‐well or 24‐well 0.4 μm polyethylene terephthalate (PET) hanging cell culture inserts (Merck) to the density of 250 000 cells/cm^2^. Passage 2 to 4 RPE cells were used for all experiments. After each passage, the RPE cells were allowed to mature (displaying TER of at least 250 Ω cm^2^) before being used for experiments. RPE characterization was performed as described in our previous publication.^21^

### 4.4 Cell culture treatments

Cp40 (yI[CVW(Me)QDWSarAHRC]mI‐NH_2_) and a scrambled peptides (yI[CSarVDWAHW(Me)QRC]mI‐NH_2_), synthesized as described,^37^ of 6 μM final concentration were used for a 7‐day treatment, with peptide‐containing media change after 3.5 days. Rapamycin (InvivoGen) of 500 nM final concentration was used for a 7‐day treatment, with daily media change containing rapamycin.

### 4.5 Immunoassays

#### 4.5.1 *Western blotting*

Cell culture samples were collected by using a cell scraper and cells were lysed using PhosphoSafe Extraction Reagent (Merck). BCA Protein Assay Kit (ThermoFisher) was used to quantify protein concentration in the samples for a consistent gel loading. Western blotting was performed using Invitrogen precast gels and a dry blotting system. Zinc stain (ThermoFisher) was used to visualize protein quantities on a gel prior to transfer and Ponceau Stain was used to visualize protein quantities on a polyvinylidene difluoride (PVDF) membrane after the transfer. PVDF membranes were blocked using 5% dried skimmed milk in tris‐buffered saline and Tween™ 20 solution (TBST). Following an overnight 4°C incubation with primary antibodies in blocking buffer, the membranes were washed in TBST and incubated with horseradish peroxidase (HRP) conjugated secondary antibodies and SuperSignal West Pico PLUS Chemiluminescent Substrate (ThermoFisher) was used for signal detection. This signal was visualized using an Amersham Imager 600 (GE Healthcare Bio‐Sciences AB) imager. The source and dilution of antibodies is shown in Table S1. Quantification of signal intensity was performed using Image Studio Lite v5.2 (LI‐COR Biosciences). Data analysis and plotting was performed using GraphPad Prism v8.3.1 (GraphPad Software).

#### 4.5.2 *Mesoscale discovery and ELISA assays*

To measure the total amount of C3b, iC3b, and C3c in tissue culture supernatant, an ELISA was established that captured these fragments using mAb bH6 (Hycult Biotech, The Netherlands; 2 μg/mL coat); C3b (Complement Technology, Tyler, Texas) was used for the standard curve. Plates were blocked with phosphate‐buffered saline (PBS), 5% nonfat milk (wt/vol), 0.1% tween 20 and samples were diluted in PBS, 1% bovine serum albumin (BSA) (wt/vol), 10 m mM EDTA. Bound C3 fragments were detected using an in‐house polyclonal rabbit anti‐human C3 (1 μg/mL of total purified immunoglobulin) followed by goat anti‐rabbit Ig‐HRPO (Jacksons Immunoresearch). The assay was developed using (o‐Phenylenediamine dihydrochloride) according to manufacturer's instructions (Sigma Aldrich) and absorbance was measured at 492 nm. Values were interpolated from the standard curve.

To measure total uncleaved C3, a Meso Scale Discovery assay was established that captured the C3a domain (Clone2898; HM2075, Hycult Biotech) and detected the C3b domain (mAb3; HM2198, Hycult Biotech). All steps were carried out at room temperature other than coating, which was at 4°C. MSD Gold 96‐well Streptavidin QUICKPLEX plates (Meso Scale Diagnostics) were blocked with PBS/10 mM EDTA, 3 wt%/vol% BSA and anti‐C3a‐biotin was captured (2 μg/mL). Supernatants were added to the wells at 1/8 dilution followed by detection with mAb3 (HM2198, Hycult Biotech; 2 μg/mL) conjugated with SULFO‐TAG NHS Ester as per manufacturers' instructions. After washing, 2X reading buffer was added to each well and electrochemiluminescence signal was immediately measured in a Meso QuickPlex SQ 120 MSD reader. Data were analyzed using the Discovery Workbench 4.0 software (MSD, Rockville, Maryland).

#### 4.5.3 *Confocal immunocytochemistry*

iPSC‐RPE cells grown on 12‐well or 24‐well 0.4 μm PET hanging cell culture inserts (Merck) were fixed with 4% paraformaldehyde for 20 minutes at room temperature; pigmentation was removed using Melanin Bleach Kit (Polysciences). The membranes were blocked using 10% donkey serum in PBS and (if needed) permeabilized using 0.3% Triton‐X100. The source and dilution of antibodies is shown in Table S1. At least six different *z*‐stacked images were acquired for each condition using an inverted Nikon A1R laser scanning confocal microscope with NIS‐Elements software. The images were taken at the exact same laser power in series with a 4x line average method. All images were deconvolved with Huygens Professional version 19.04 (Scientific Volume Imaging, The Netherlands, http://svi.nl). The deconvolution was performed using the Classic Maximum Likelihood Estimation with an absolute background reduction value identical to all images in a full set. Colocalization was determined using Manders' coefficient.^62^ The volumetric data were calculated from all objects with a fluorescence signal intensity above the subtracted background. All calculated volumes were normalized by the number of cell nuclei. Data analysis and plotting was performed using GraphPad Prism v8.3.1 (GraphPad Software).

### 4.6 Other assays

#### 4.6.1 *Cathepsin D activity assay*

Cathepsin D kinetic activity assay was performed using cell lysates diluted in 50 mM sodium acetate (pH 4.0). Following addition of 0.25 μg total protein and 80 μM Cathepsin D & E substrate (fluorogenic) (Enzo), the signal (excitation 320 nm, emission 400 nm) was measured over 30 minutes and average reaction velocity determined from an initial linear change phase. Pepstatin A (Enzo) was used to subtract any activity originating from any unrelated breakdown of substrate. Data analysis and plotting was performed using GraphPad Prism v8.3.1 (GraphPad Software).

#### 4.6.2 *Lysotracker green DND‐26 assay*

Fully confluent RPE cells were grown on Nunc 4‐well cell culture treated dishes with Nunclon Delta surface (ThermoFisher). The lysotracker probe stock solution was warmed up to room temperature and diluted to 1 μM final working concentration in warm RPE cell culture medium. Growth medium was removed from cell culture dishes and the diluted lysotracker probe added to cells and incubated for 1 hour at optimal growth conditions. Following the incubation, the probe was removed and replaced with warm growth medium (wash) and a final volume of growth medium. Cells were imaged immediately using Nikon A1R confocal microscope at the exact same laser power in series with a 4x line average method. Average image intensity was quantified from a maximum intensity projection analysis of 6 x20 images using Image J program (NIH).

### 4.7 Transmission electron microscopy

iPSC‐RPE cells grown on 12‐well or 24‐well 0.4 μm PET hanging cell culture inserts (Merck) were fixed in 2% glutaraldehyde in 0.1 M cacodylate buffer. After rinsing in buffer, the samples were postfixed in 2% osmium tetroxide containing 0.8% potassium ferricyanide, rinsed in deionised water then dehydrated through a graded series of acetone. Tissue was infiltrated with epoxy resin (TAAB medium) and polymerized at 60°C for 36 hours. Ultrathin sections (70 nm) were cut on a Leica ultramicrotome and picked up on copper grids. The grids were stained with uranyl acetate and lead citrate before being imaged on a 120 kV HT7800 TEM (Hitachi). At least 10 cells were imaged per sample, selected randomly along the length of the insert, provided that the cell was intact with a clearly demarcated cell membrane. Objects identified in the images were measured using ImageJ with tools calibrated to the burned‐in scale bar.

### 4.8 Quantitative RT‐PCR

ReliaPrep RNA Miniprep System (Promega) was used to isolate RNA, which was then transcribed to cDNA by GoScript Reverse Transcriptase (Promega). To perform a quantitative PCR, GoTaq qPCR mix (Promega) was used with QuantStudio 7 Flex Real‐Time PCR thermocycler (ThermoFisher). The data was logged and processed with QuantStudio Real‐Time PCR Software v1.3. Data analysis and plotting was performed using GraphPad Prism v8.3.1 (GraphPad Software). The sequence of DNA oligonucleotides is shown in Table S2.

## CONFLICT OF INTEREST

D.S. declared Consultant/Advisory role Alcon, Roche, Gyroscope and research funding from Alcon, Roche. M.D. declared research funding but not directly related to this work. D.K. declared Consultant/Advisory, stock interest with Gyroscope Therapeutics; honoraria from Gyroscope Therapeutics, Alexion Pharmaceuticals, Novartis, Apellis, Sarepta. J.D.L. is the founder of Amyndas Pharmaceuticals, which is developing complement inhibitors for therapeutic purposes and is also the inventor of the compstatin technology licensed to Apellis Pharmaceuticals (ie, 4(1MeW)7W/POT‐4/APL‐1 and PEGylated derivatives such as APL‐2/Pegcetacoplan). J.D.L. is inventor of patent or patent applications that describe the use of complement inhibitors for therapeutic purposes, some of which are developed by Amyndas Pharmaceuticals. C.H. declared patent ownership of factor H polymorphisms in the diagnosis and therapy of inflammatory diseases such as age‐related macular degeneration and consultant for GlaxoSmithKline, Admirx Inc, Freeline Therapeutics, Gyroscope Therapeutics and research funding from Ra Pharmaceuticals. All of the other authors declared no potential conflicts of interest.

## AUTHOR CONTRIBUTIONS

E.C.: experimental design and execution, data analysis, manuscript and figure preparation; M.K.‐A.: data acquisition, data analysis, figure preparation; L.X.: data acquisition, data analysis, manuscript and figure preparation; D.H.: data acquisition, data analysis, contributed to figure preparation; M.M.‐M., K.W., M.D., P.W.: data acquisition, data analysis; D.S., J.A.‐A., L.A., D.K.: experimental design, fund raising; J.D.L.: provided reagents, experimental design; V.I.K.: experimental design, data analysis, contributed to manuscript preparation, fund raising; C.H.: provided reagents, experimental design, data analysis, contributed to manuscript preparation, fund raising; M.L.: experimental design, data analysis, manuscript preparation, fund raising, overall project management. All authors approved the final version of the manuscript.

## Supporting information


**Figure S1** **Assessment of autophagy‐related protein levels and autophagic flux in low‐ and high‐risk RPE cells. A)** Western blotting quantification shows a significant increase of LC3‐II, p62 and ATG12‐ATG5. All quantification data were normalised to the total protein blots. Data shown as mean +/− SEM (data from at least 4 replicates); **B)** Immunocytochemistry data show a significant increase of intracellular LC3 and p62 in high‐risk RPE cells. Data were calculated as volume/cell. Data shown as mean +/− SEM (data from at least 6 different images per cell line, all experiments performed in biological triplicates); **C**) Western blot representative examples (n = 3) showing reduced autophagic flux in high‐risk RPE cells reflected in no changes in LC3‐II expression in response to Rapamycin and Bafilomycin A1 treatments.
LR = low‐risk RPE cells; HR = high‐risk RPE cells. Statistical significance was assessed using an unpaired *t*‐test.

**Figure S2** **Induction of autophagy via Rapamycin application has no beneficial effects on high‐risk RPE cells**. **A**) Rapamycin application has no impact on cell lysate derived cathepsin D activity of high‐risk RPE cells; **B**) Representative TEM examples of high‐risk RPE cells before and after application of Rapamycin together with quantification analysis. Data shown as mean +/− SEM, n = 10‐15; **C**) Quantification of melanosomes and lysosome‐like‐ vesicles in high‐risk RPE cells before and after rapamycin treatment. Data shown as mean +/ SEM, n = 10‐15; **D**) Quantification of stress vacuoles in high‐risk RPE cells before and after rapamycin treatment. Data shown as mean +/ SEM, n = 10‐15.
LR = low‐risk RPE cells; HR = high‐risk RPE cells. Statistical significance was assessed using an unpaired *t*‐test, * *P* < 0.05, ** *P* < 0.01, *** *P* < 0.001.

**Figure S3** **High‐risk RPE cells are characterised by higher C3 turnover reflected in the significant increase in the ratio of C3 fragments to native C3.** A) Quantitative RT‐PCR analysis of key components of complement pathway in low‐ and high‐risk RPE cells; **B**) Measurement of activated C3 (C3b, iC3b, C3c) in supernatants of low‐ and high‐risk RPE cells; **C**) Measurement of native C3 (comprising both C3a and C3b domains) in supernatants of low‐ and high‐risk RPE cells; **D**) Ratio of C3 fragments to native C3 in low‐and high‐risk RPE cells.
A‐D) Data shown as mean +/− SEM, n = 3.
LR = low‐risk RPE cells; HR = high‐risk RPE cells. Statistical significance was assessed using an unpaired *t*‐test, * *P* < 0.05, ** *P* < 0.01, *** *P* < 0.001.

**Figure S4** **CP40 treatment of high‐risk RPE cells reduced C3 turnover reflected in the significant reduction in the ratio of C3 fragments to native C3**. **A**) Quantitative RT‐PCR analysis of key components of complement pathway in high‐risk RPE cells treated with the scramble and Cp40 peptide; **B**) ELISA measurement of activated C3 in scrambled and CP40 treated high‐risk RPE cells; **C**) Levels of native C3 in supernatants of scrambled and CP40 treated high‐risk RPE cells; **D**) Ratio of C3 fragments to native C3 in scrambled and CP40 treated high‐risk RPE cells.
A‐D) Data shown as mean +/ SEM, n = 3.
HR = high‐risk RPE cells, SCR‐ scrambled peptide. Statistical significance was assessed using an unpaired *t*‐test, * *P* < 0.05, ** *P* < 0.01, *** *P* < 0.001.

**Figure S5** **Cp40 treatment restores lysosomal function in high‐risk RPE cells.** Cp40 treatments results in an increase in CTSD expression (**A**), cell lysate derived Cathepsin D activity (**B**) and reduction of lysosome‐like‐vesicle size (**C**) in high‐risk RPE cells.
A‐C) Data shown as mean +/ SEM, n = 3.
HR = high‐risk RPE cells, SCR‐ scrambled peptide, ** *P* < 0.01, *** *P* < 0.001.

**Figure S6** Representative TEM images showing a considerable increase in stress vacuoles (shown by white arrows) in high‐risk RPE cells treated with *C3* shRNA, when compared to the scramble shRNA control.

**Table S1** 

**Table S2** 

## Data Availability

The data that support the findings of this study are available on request from the corresponding author.
