# Supplementary material for: Complement modulation reverses pathology in Y402H‐retinal pigment epithelium cell model of age‐related macular degeneration by restoring lysosomal function
Source: Stem Cells Transl Med. 2020 Aug 20;9(12):1585–603. doi: 10.1002/sctm.20-0211 (PMC7695639; doi:10.1002/sctm.20-0211)
Supplement: Supplementary file 7 — Table S1 [file SCT3-9-1585-s007.docx]

# **Table S1**

| **Target** | **Dilution** | **Supplier** | **Stock Keeping Unit** |
| --- | --- | --- | --- |
| ACTB | WB: 1:500 | Santa Cruz Biotechnology | sc-47778 |
| ApoE | ICC: 1:1000 | Merck | AB947 |
| ATG5 | WB: 1:500 | Sigma-Aldrich | A0856 |
| BECN1 | WB: 1:500 | Abcam | ab210498 |
| BiP | WB: 1:500 | Cell Signaling Technology | 3177T |
| C3 | WB: 1:500 | Abcam plc | ab48611 |
| C3 | ELISA: 0.85 mg/ml | In-house | In-house |
| C3b | ICC: 1:50 | Hycult Biotech | HM2168 |
| C5b-9 | ICC: 1:200 | Agilent | M0777 |
| CD63 | WB: 1:500  ICC: 1:150 | Abcam plc | ab59479 |
| CTSD | WB: 1:2000  ICC: 1:200 | Sigma-Aldrich | C0715 |
| CTSD | ICC: 1:50 | Sino Biological | 12517-R003 |
| GAPDH | WB: 1:500 | Santa Cruz Biotechnology | sc-47724 |
| iC3b | ICC: 1:50 | Bio-Rad | MCA2607 |
| IgG (goat) | ICC: 1:200 | Thermo Fisher Scientific | A-21447 |
| IgG (goat) | WB: 1:2000 | Agilent | P0449 |
| IgG (mouse) | ICC: 1:1000 | Jackson ImmunoResearch | 715-095-151 |
| IgG (mouse) | WB: 1:2000 | Agilent | P0260 |
| IgG (rabbit) | ICC: 1:1000 | Jackson ImmunoResearch | 711-295-152 |
| IgG (rabbit) | ELISA: 1:200 | Jackson ImmunoResearch | 111-036-144 |
| IgG (rabbit) | WB: 1:2000 | Agilent | P0399 |
| LAMP1 | WB: 1:500 | Developmental Studies Hybridoma Bank | H4A3 |
| LAMP2 | ICC: 1:100 | Antibodies.com | A86605 |
| LAMP2 | WB: 1:500  ICC: 1:100 | Abcam plc | ab199946 |
| LGALS8 | ICC: 1:100 | Biorbyt Ltd | orb216142 |
| MAP1LC3B | WB: 1:500  ICC: 1:200 | Cell Signaling Technology | 3868 |
| p62 | WB: 1:500  ICC: 1:200 | BD Biosciences | 610832 |
| p-S6 (Ser235/236) | WB: 1:500 | Cell Signaling Technology | 2211 |
| Rab5 | WB: 1:500 | Abcam | ab218624 |
| ZO1 | ICC: 1:50 | St John's Laboratory | STJ140055 |
| ZO1 | ICC: 1:50 | Invitrogen | 61-7300 |
