# Supplementary material for: Complement modulation reverses pathology in Y402H‐retinal pigment epithelium cell model of age‐related macular degeneration by restoring lysosomal function
Source: Stem Cells Transl Med. 2020 Aug 20;9(12):1585–603. doi: 10.1002/sctm.20-0211 (PMC7695639; doi:10.1002/sctm.20-0211)
Supplement: Supplementary file 8 — Table S2 [file SCT3-9-1585-s008.docx]

# **Table S2**

| **Target** | **Direction** | **Sequence** |
| --- | --- | --- |
| *C3* | Forward | TCCGGAACTCGTCAACATGG |
|  | Reverse | CAATCGGAATGCGCTTGAGG |
| *C5* | Forward | AACCATGGGCCTTTTGGGAA |
|  | Reverse | GACATATGTTTGCTCCTGTCCC |
| *C6* | Forward | CAGGGGAATTCCTGGACACC |
|  | Reverse | TCTGAATGATGGCTACAGTCTTCT |
| *C7* | Forward | AGTGGGACTTCTATGCCCCT |
|  | Reverse | GCACTGACCTGAAAAGCACC |
| *C8γ* | Forward | TTCCGAAAGCTGGATGGGAT |
|  | Reverse | CCAGGTACAGGACAGCGAAA |
| *C9* | Forward | GAACAGCAGGCTATGGGATCA |
|  | Reverse | CACGTTCCAAGGTCTTCGGT |
| *GAPDH* | Forward | TGCACCACCAACTGCTTAGC |
|  | Reverse | GGCATGGACTGTGGTCATGAG |
